# Intersectional Hybrids between Darrow’s Blueberry (*V. darrowii Camp*) and Lingonberry (*V. vitis-idaea* L.)

**DOI:** 10.3390/plants13111572

**Published:** 2024-06-06

**Authors:** Mark K. Ehlenfeldt, Nahla Bassil, Ryan King, Juan Zalapa, Fernando de la Torre, James L. Luteyn

**Affiliations:** 1 USDA-ARS, Philip E. Marucci Center for Blueberry and Cranberry Research and Extension, Chatsworth, NJ 08019, USA; 2 USDA-ARS, National Clonal Germplasm Repository (NCGR), 33447 Peoria Road, Corvallis, OR 97333, USA; nahla.bassil@usda.gov (N.B.);; 3 USDA-ARS, Vegetable Crops Research Unit, Department of Plant and Agroecosystem Sciences, University of Wisconsin, Madison, WI 53705, USA; 4 Vegetable Crops Research Unit, Department of Plant and Agroecosystem Sciences, University of Wisconsin, Madison, WI 53705, USA; 5 New York Botanical Garden, Bronx, NY 10458, USA; jim.luteyn@gmail.com

**Keywords:** allotetraploid, endosperm balance number (EBN), genome strength, polyploid

## Abstract

An initial cross of *V. darrowii* ‘Johnblue’ (Darrow’s blueberry) × *V. vitis-idaea* ‘Red Sunset’ (lingonberry) produced more than 30 true intersectional diploid hybrids as confirmed by molecular markers. The most vigorous of these hybrids was extensively evaluated. This hybrid, US 2535-A, was floriferous and morphologically intermediate to the respective parents. Examination of pollen suggested low male fertility. Numerous crosses using the hybrid as a female reflected similarly low fertility and potential crossing barriers. Stylar examination suggested blockage of pollen tube growth in self-pollinations and significantly retarded growth in backcross pollinations. Nonetheless, two confirmed hybrid offspring were produced using the F_1_ hybrid as a female in crosses with *V. vitis-idaea* and *V. darrowii*, respectively. In a second set of crosses utilizing additional *V. darrowii* and *V. vitis-idaea* genotypes, another 23 verified hybrids in seven parental combinations were produced. Hybrids such as the ones presented offer the potential for generating de novo interspecific fruit types in blueberry and/or broadening the adaptation of lingonberry.

## 1. Introduction

Darrow’s blueberry, *Vaccinium darrowii* Camp, and lingonberry, *V. vitis-idaea* L., are two *Vaccinium* species with widely divergent biologies. While *V. darrowii* is a southern-adapted blueberry species of section *Cyanococcus*, *V. vitis-idaea* (lingonberry) is a low-growing circumboreal evergreen species of section *Vitis-idaea.* Morphologically, these two species differ widely.

*V. darrowii* is a rhizomatous, colonial species found in scrubby flatlands in a range covering Florida, Georgia, Alabama, and parts of Louisiana. *V. darrowii* is evergreen with small waxy leaves and forms small bushes seldom over 90 cm high. Its flowers are urceolate to cylindrical, 4–6 mm long, and typically white but often tinged with pink. Plants often flower profusely and produce numerous small, waxy, and blue berries 8–10 mm in diameter. *V. darrowii* is a diploid (2*n* = 2*x* = 24) and is particularly notable for the role it has played in the development of the southern highbush blueberry [1]. *V. darrowii* has no flower-bud chilling requirement, and in the 1950s, a native *V. darrowii* clone designated ‘Fla 4B’ was used to introduce lower flower-bud chilling requirements into northern highbush blueberry (*V. corymbosum*) [2]. The *V. darrowii* germplasm was introduced via the use of 2*n* gametes and resulted in *V. corymbosum*-derived cultivars with approximately half the chilling requirement of typical northern highbush blueberry. Along with lower chilling requirements, *V. darrowii* transferred plant vigor, an expanded range of fruit volatiles, and increased fruit firmness. Fla 4B has subsequently been determined to have a genetic ancestry that is 68% *V. darrowii* and 32% *V. fuscatum* [3]. *V. darrowii* is considered by many researchers to be the most ancestral North American species [4] and is possibly the most cross-compatible and most genotypically plastic.

In contrast, *V. vitis-idaea* (lingonberry) is a low-growing circumboreal evergreen species of the monospecific section *Vitis-idaea.* Lingonberry is rhizomatous and semi-woody and possesses dark green, leathery, obovate leaves. Its flowers have bell-shaped corollas and are produced singly or as clusters. Lingonberry is highly self-fertile. Plants produce red, shiny fruit, typically 7–11 mm in diameter. *V. vitis-idaea* is a diploid with 2*n* = 2*x* = 24.

Lingonberry fruit is harvested commercially in Scandinavian countries, Canada, and Alaska from both cultivated and wild stands. There has been much interest in growing lingonberry in southern temperate areas of North America; however, such attempts have failed due to a lack of proper adaptation and susceptibility to root pathogens that may kill entire stands (MKE personal observation).

Reports of hybrids of lingonberry with other *Vaccinium* species are limited. Natural hybrids of lingonberry and *V. myrtillus* (section *Myrtillus*) termed *V. intermedium* were described by Ritchie [5,6]. These hybrids apparently occurred repeatedly; however, their vigor and fertility were reported to be low.

At the diploid level, several successful efforts have hybridized 2*x V. vitis-idaea* (lingonberry) and 2*x V. macrocarpon* (cranberry) [7,8,9]. Lingonberry was also successfully hybridized with the Hawaiian species 2*x V. reticulatum* section *Macropelma* (Ōhelo berry) [10]; however, in both instances, only limited fertility was reported to exist, and no reports were found of advanced materials.

At the tetraploid level, Morozov [11] succeeded in hybridizing 4*x V. uliginosum* (bog bilberry) and a 4*x* genotype of lingonberry. Unlike diploids, these F_1_ hybrids were fertile and were subsequently crossed to 4*x* cranberry, 4*x* highbush blueberry, 4*x* half-high blueberry, and backcrossed to 4*x* lingonberry. Some of these materials were generationally advanced by Marozau and Baranov [12], who described trispecific hybrids of (*Vaccinium uliginosum* L. × *V. vitis-idaea* L.) × *Oxycoccus macrocarpus* (Aiton) Pursh (cranberry). Morosov [11] also reported successful primary hybridization of 4*x* lingonberry × 4*x* lowbush blueberry (*V. angustifolium*). More recently, Ehlenfeldt and co-workers [13] reported the production of fertile 4*x* hybrids of 4*x V. meridionale* (Andean blueberry) with 2*x V. vitis-idaea* via 2*n* gametes.

In the course of investigating the crossability of multiple *Vaccinium* species belonging to different *Vaccinium* sections and different ploidy levels, we succeeded in hybridizing *V. darrowii* with *V. vitis-idaea* and generating multiple progenies. We report here on the evaluations of the most vigorous of these hybrids as investigated by post-pollination stylar staining, assessment of pollen quantity and quality, and female fertility determination (based on number of pollinations, fruit set, seed number, and seedling success). Additionally, we report on further hybridization of these two species and extensive evaluations of all generated hybrids, including ploidy determinations, genotyping using SSR markers, and other phenotypic assessments.

## 2. Results

### 2.1. Initial Crosses

The cross of *V. darrowii* ‘Johnblue’ × *V. vitis-idaea* ‘Red Sunset’ was initially performed on a modest scale to evaluate intersectional compatibilities. This cross was part of a larger study evaluating intersectional compatibilities in a much wider range of *Vaccinium* germplasm. In the initial test pollinations, 34 pollinations produced 16 fruits. Among the seeds extracted from these fruits, none were rated as fully ‘good’. Twenty (20) seeds were rated as ‘good-fair’, 78 as ‘fair’, and 15 as ‘fair-poor’. In the accompanying extraction notes, even the ‘good-fair’ seeds were described as small, relatively thin, and flattish.

### 2.2. Morphology

**Figure 1:**
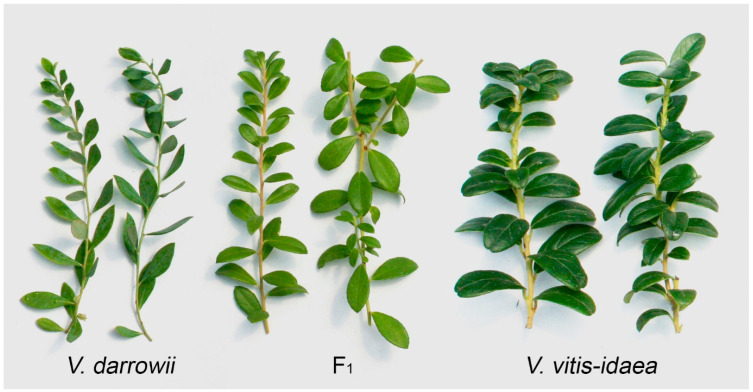
Foliage (L to R, pairs of branches) of *V. darrowii* ‘Johnblue’, the F_1_ hybrid US 2535-A, and *V. vitis-idaea* ‘Red Sunset’.

In the first year, the *V. darrowii* × *V. vitis-idaea* hybrids grew slowly. During this period, plant morphology was insufficiently distinct to identify any potential hybrid plant with any certainty. The cross family as a group was tremendously variable and well within what we have previously observed in 100% *V. darrowii* populations. All plants were evaluated for vigor on a scale of 1–4 based on their ability to grow quickly and large. Among thirty-five individual seedlings, three were considered large and vigorous, twenty-two were of intermediate vigor and stature, seven were considered petite, and three died prematurely. Mature leaf sizes on vigorous clones averaged 18 mm (L) × 8 mm (W), with the largest observed leaves approximately 24 mm (L) × 8.5 mm (W). Mature leaves on petite clones were typically 7–8 mm long and ranged from 3–6 mm wide. All of the putative hybrids appeared to have medium light-green colored foliage, unlike the female parent ‘Johnblue’, which was distinctly blue-green, especially on young tissue (Figure 1). This coloration, however, was not considered diagnostic since both leaf expansion and surface wax erosion on *V. darrowii* can alter the leaf surface color to a similar greenish shade.

By the second season, several plants began to grow with more vigor, and one plant in particular, subsequently designated US 2535-A, began to exhibit morphology that was recognizably intermediate to the two parents (Figure 1). The hybridity of US 2535-A was subsequently confirmed using molecular markers.

US 2535-A hybrid leaves were light green in color and intermediate in shape to the two parents. US 2535-A was notable for its larger, more rounded leaves than ‘Johnblue’, and a noticeably undercurled leaf margin as opposed to that seen on ‘Johnblue’. Leaf venation in US 2535-A was conspicuous and pinnate, with the three main lateral veins anastomosing near their margins like that of lingonberry. The leaf apices were acute overall but also bluntly mucronate (ending abruptly in a sharp point). The leaves were slightly curved towards the apex, and the overall blade was slightly concave, not flat. The leaf margins were crenate (having a round-toothed or scalloped edge), these being about eight per side and each tipped with a deciduous, reddish, tiny, glandular hair. Petioles were slightly flattened on both surfaces and were puberulent (covered with fine pubescence). The twigs and stems were also puberulent. The lower leaf surfaces had scattered glandular hairs.

**Figure 2:**
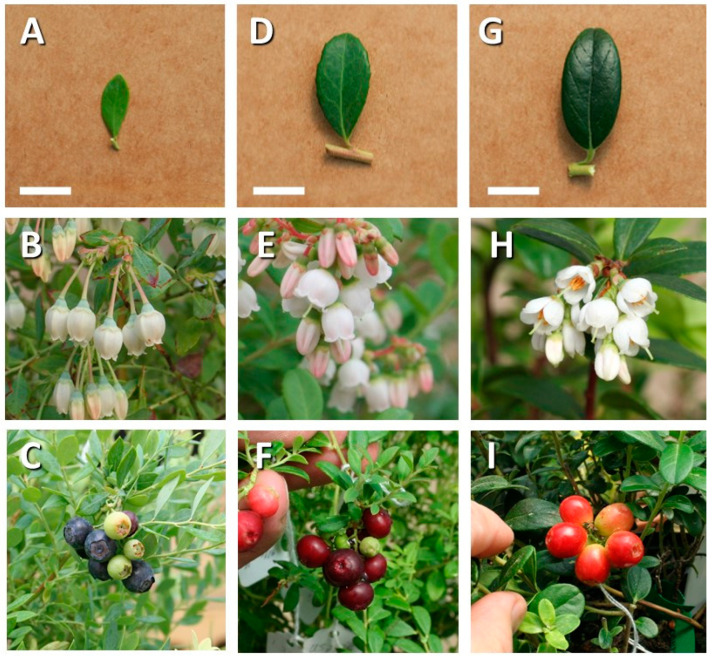
Leaves, flowers, and fruits of *V. darrowii* ‘Johnblue’ (**A**–**C**), US 2535-A (**D**–**F**), and *V. vitis-idaea* ‘Red Sunset’ (**G**–**I**). Leaf scale = 1 cm; flowers and fruits, no scale.

Flower morphology of the US 2535-A hybrid was distinctive from either parent and was the first indicator that US 2535-A was a true hybrid (Figure 2). Flower buds were white with pink blush and were bluntly angled or ribbed. Mature corollas were white, ovate-campanulate (bell-shaped), nearly terete (cylindrical or slightly tapering and without substantial furrows or ridges), and slightly constricted at the throat, with four or five lobes that were spreading-reflexed when mature. Styles were noticeably exserted, a distinct feature inherited from lingonberry. The mature fruits were slightly flattened, spherical, and terete, so the top of the ovary was inconspicuous. Fruits were 9–11 mm in diameter under ideal circumstances, decreasing to much smaller sizes in less successful pollinations. Fruits were non-waxy and relatively dull, with the color at maturity ranging from dark purple-red (RHS 59A/Pantone 1815C) to dark purple (RHS N79A/Pantone 504C) (Figure 2).

**Figure 3:**
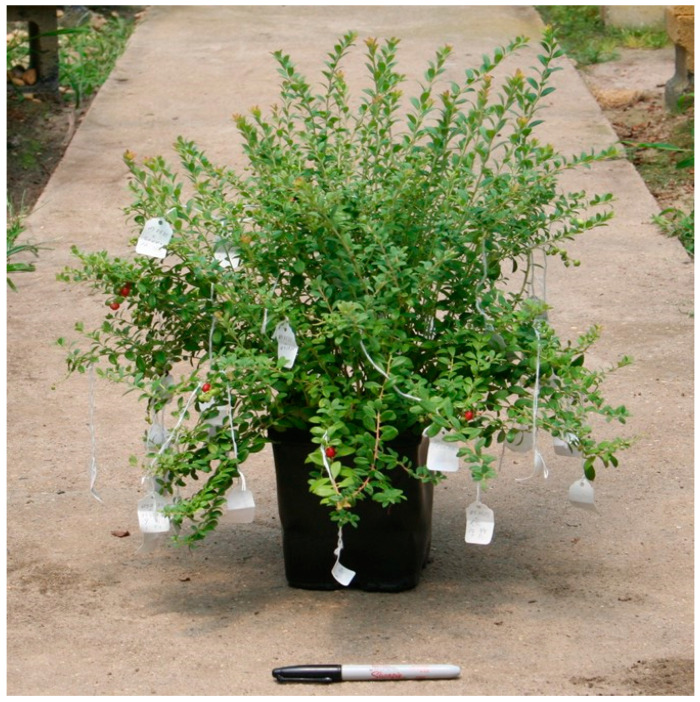
A plant of US 2535-A (*V. darrowii* ‘Johnblue’ × *V. vitis-idaea* ‘Red Sunset’).

US 2535-A produced shoots in long growth flushes similar to *V. darrowii*. The most proximal growth was relatively coarser with larger leaves, proceeding to finer growth as shoots extended in length. Unlike lingonberry, the hybrid exhibited no indication of rhizomatous growth. The plant had a full, vigorous, bushy structure (Figure 3), with an estimated potential to form upright plants similar in size to *V. darrowii* clones.

### 2.3. Ploidy Determinations

In several other 2*x* × 2*x* intersectional crosses of *V. vitis-idaea* × *V.* spp. in our program, we have observed triploid production that appeared to be due to the functioning of 2*n* gametes from ‘Red Sunset’. Because of this, a sampling of putative F_1_ hybrids was assayed for ploidy verification using flow cytometry. The 31 clones assayed, including US 2535-A, were found to all be diploid, 2*n* = 2*x* = 24, the expected ploidy from the parental 2*x* × 2*x* cross. The mean DNA content across the 31 hybrids was 1.09 pg (s.d = 0.037 pg, range = 0.99–1.17 pg) (Table 1). All values were within ±10% of the predicted midparent value.

### 2.4. Paternity Analysis with SSR of F_1_s and Backcrosses

**Figure 4:**
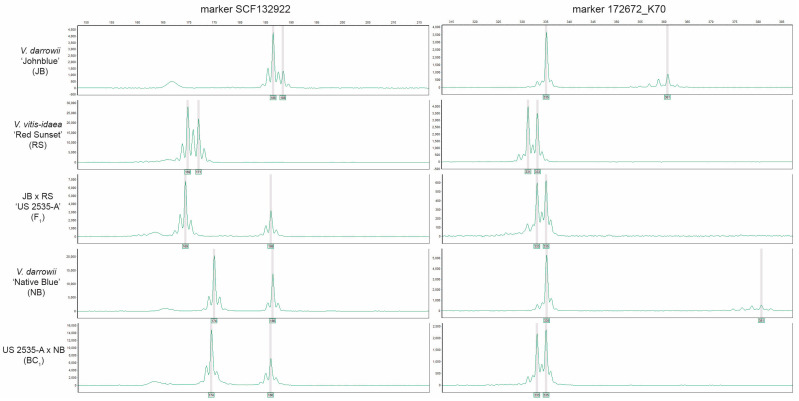
Simple sequence repeat (SSR) allelic signatures of *Vaccinium* progenitors (*V. darrowii* ‘Johnblue’ (JB) and ‘Native Blue’ (NB), as well as *V. vitis-idaea* ‘Red Sunset’ (RS)); F_1_ and BC_1_ hybrids for markers SCF132922 and 172672_K70.

Genetic analysis was performed using an SSR marker set to further substantiate the hybridity of US 2535-A and the additional F_1_ hybrids. SSR fragment scores (in bps) were determined at each marker for every plant, including the parents and putative offspring (Table 2). Polymorphic loci were targeted by PCR using six primers, modified with HEX-M13 sequences to produce SSR fragments. Two such markers, SCF132922 and 172672_K70, and their separation after electrophoresis are shown here (Figure 4). For diploid individuals, two major fragments (X-axis, bps) are expected if the individual carries two distinct alleles at the targeted locus. The signal of the allele fragment is measured by fluorescence intensity (Y-axis). The height of the SSR fragment was scored with a minimally acceptable signal being at least 200 intensity.

The SSR fragment segregations agreed with the expectation that all plants in this study were diploid. The six markers used to determine hybridity were the following: SCF275d, SCF804, SCF9815, SCF37628, SCF132922, and 172672K70 [14]. Based on the data presented in Table 2, US 2535-A, as well as 28 other plants, were unequivocally F_1_ hybrids between *V. darrowii* ‘Johnblue’ × *V. vitis-idaea* ‘Red Sunset’ since each offspring inherited a unique allele from both ‘Johnblue’ and ‘Red Sunset’. Seven SSR reactions failed to produce any fragment signatures due to poor-quality DNA based on low-quality leaf tissue for those samples rather than lack of hybridity.

In the initial seed production, only 20 seeds had been rated as ‘good-fair’, and the fact that we have almost 30 confirmed hybrids suggests that even some of the seeds that were only rated as fair gave rise to plants. These plants continue to be grown to assess plant phenotypes, flower morphology, and potential fertility.

### 2.5. Male Fertility

**Figure 5:**
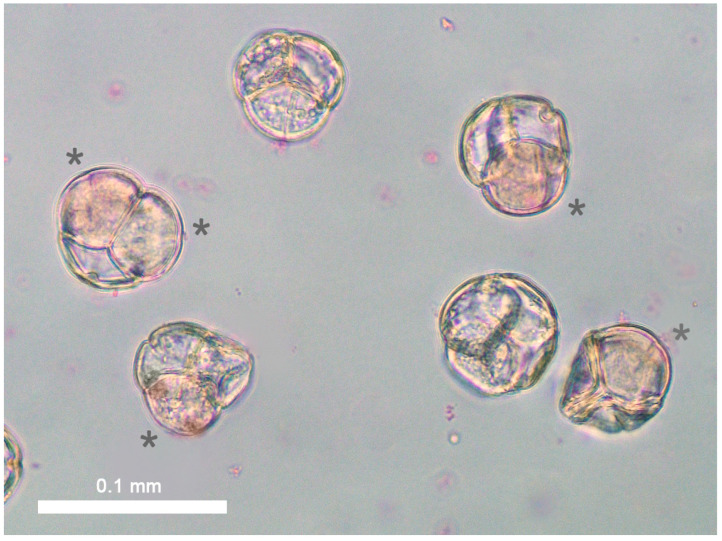
Pollen tetrads of US 2535-A (200×). Asterisks highlight potentially viable pollen microspore segments. Scale equals 0.1 mm.

US 2535-A exhibited fair pollen shed; however, microscopic examination revealed poor pollen quality with no perfect tetrads observed. In many cases, all four microspores of the tetrad appeared defective; however, in several instances, single microspores within a tetrad appeared essentially normal (Figure 5). The frequency of such tetrads was evaluated and estimated to be approximately 20%.

When tested for growth, US 2535-A pollen appeared to hydrate normally but did not germinate in vitro, reflective of the observed condition of pollen tetrads (Figure 5). None of the examined styles that received US 2535-A pollen (‘Johnblue’, ‘Red Sunset’, or US 2535-A selfed) showed any pollen tubes traveling down the styles after staining with aniline blue and careful dissection.

The low–moderate pollen shed of US 2535-A and the cautiously optimistic evaluation of pollen suggested that slight male fertility might exist given a sufficient number of pollinations. We, therefore, undertook a series of backcrosses of the general type *V. vitis-idaea* × US 2535-A and *V. darrowii* × US 2535-A. These unemasculated backcrosses onto ‘Red Sunset’ and ‘Erntedank’ lingonberry produced 32 seeds/33 pollinations and 1 seed/22 pollinations, respectively (Table 3). Unemasculated backcrosses onto the *V. darrowii* cultivars, ‘Johnblue’ and ‘Native Blue’, produced 13 seeds/33 pollinations and 0 seed/15 pollinations, respectively. These *V. vitis-idaea* and *V. darrowii* crosses produced 20 and seven plants, respectively, all of which were identified as selfs based on SSR testing.

### 2.6. Female Fertility

Simultaneous to other evaluations, US 2535-A was backcrossed as female to three different *V. vitis-idaea* cultivars and exhibited only minimal success. US 2535-A set one seed each in crosses with ‘Red Sunset’ (one ‘good’ seed per 53 pollinations) and with ‘Sanna’ (one ‘fair’ seed per 12 pollinations). Similar limited success was seen in the backcross US 2535-A × 2*x V. darrowii* ‘Native Blue’ (one seed/24 pollinations). The US 2535-A × 2 × *V. darrowii* ‘Native Blue’ seed and the US 2535-A × ‘Sanna’ seed germinated and subsequently underwent paternity testing. Both were confirmed as BC_1_ hybrids. The seed of US 2535-A × ‘Red Sunset’ failed to grow (Table 3 and Table 5).

**Figure 6:**
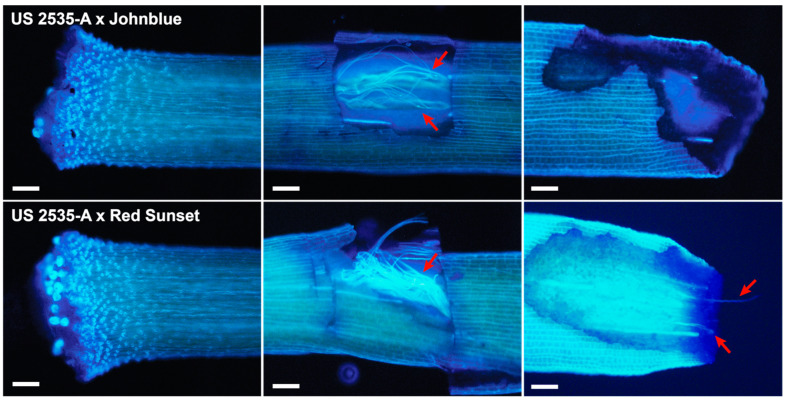
Styles of US 2535-A pollinated with *V. darrowii* ‘Johnblue’ and *V. vitis-idaea* ‘Red Sunset’. The styles of US 2535-A pollinated with ‘Johnblue’ showed elongation through mid-style (red arrows) but no presence at the style base; the styles of US 2535-A pollinated with ‘Red Sunset’ showed pollen tubes at mid-style and slight penetration of pollen tubes to style base (red arrows). Scale bar = 100 μm.

Subsequent stylar examinations with ‘Johnblue’ and ‘Red Sunset’ pollen showed that US 2535-A accepted ‘Johnblue’ and ‘Red Sunset’ pollen, but the pollen displayed arrested growth within the style. ‘Johnblue’ and ‘Red Sunset’ pollen tubes were detected approximately 50% of the distance down the style; however, pollen tubes were either not present at the base of the style (the case for ‘Johnblue’ pollen) or were present only as very tiny fraction of the pollen applied (the case for ‘Red Sunset’ pollen)—typically one to three pollen tubes were observed after detecting modest pollen tube presence at 50% length (Figure 6). This pollen tube inhibition corroborates the low seed set noted in crosses.

### 2.7. F_1_ Cross Analogs

To follow up on the initial hybrid production, we repeated the initial type of cross with an expanded range of genotypes (Table 4). We only considered ‘good’, ‘good-fair’, and ‘fair’ seeds as likely to be viable. Across all combinations, we observed 23% ‘good’, 65% ‘good-fair’, and 10% ‘fair’ seeds. Seed morphology for most, except the ‘good’ seed, was that which we had come to expect from *V. darrowii* × *V. vitis-idaea* crosses, with observably reduced size but with good color and the retention of the appearance of semi-plumpness. This appearance appears to be associated with female-excess-type seed development [15,16]. In general, ‘Johnblue’ had the highest average success rate as a female in crosses with *V. vitis-idaea,* with 3.0 s/poll. vs. 1.1 s/poll. for ‘Native Blue’. ‘Native blue’ crosses had the highest levels of seeds rated as ‘good’ at 83% (vs 8% for ‘Johnblue’). Although it is unknown if ‘good’/normal seeds gave rise to selfs or if some of these might also have been hybrid, what can be determined is that ‘Native Blue’ also had the highest number of verified F_1_ hybrids per extracted seed (32% vs. 7% for ‘Johnblue’).

In all, we obtained 31 additional genetically confirmed F_1_ hybrids between *V. darrowii* and *V. vitis-idaea* from seven different parental combinations (Table 5). Single asterisks mainly present in column ‘SCF132922’ for ‘Native Blue’ × ‘Sanna’ F_1_ individuals (Table 4) indicate a shortfall in the SSR reaction to show the second allele that is expected from the second parent, called a ‘dropped allele’. Three sources of error that can explain dropped alleles in SSR reactions are stuttering patterns, large-allele dropout, and null alleles [17]. For example, in the case of null allele dropout, a mutation at the polymorphic locus can disrupt proper primer annealing, interrupt amplification, and cause the allele to present as ‘null’. On the other hand, large-allele dropout surfaces when amplification occurs preferentially on the smaller of two alleles, which can misidentify a heterozygote as a homozygote. Dropped alleles, most likely large-allele dropouts, were observed at two of the six SSRs used in this study, mainly among the progeny of ‘Native Blue’ × ‘Sanna’ (Table 5).

## 3. Discussion

The ability to produce an intersectional hybrid between a blueberry species, in this case, *V. darrowii*, and red-fruited non-blueberry species, *V. vitis-idaea*, is notable. The fact that numerous hybrids were readily generated in the first cycle of crossing with only modest numbers of pollinations suggests that the effective genetic distance between blueberry and lingonberry is modest. However, the substandard development of the seed from such crosses suggests that embryonic development programs are not fully compatible and are reflective of the evolutionary and geographic divergence between these two species. The small seed seen in these crosses is perhaps suggestive of female-excess type morphology [15,16] and is thus suggestive of *V. vitis-idaea* having a lower genome strength than *V. darrowii* [18], even though, somatically, these species appear to be compatible at the diploid level.

Many of the true hybrids have appeared to have a juvenile period during which they were slow to develop. The vigor of the US 2535-A, however, suggests that at least some combinations of these two parents have the potential to be significantly heterotic. The repeat of this type of cross on a larger scale with additional female and male genotypes will likely allow the selection of additional types with superior vigor.

To this end, we repeated our initial combination with several other *V. darrowii* genotypes and *V. vitis-idaea* cultivars. In these crosses, we observed aspects of seed morphology that mirrored the initial cross. It is notable that the pollinations produced both normal-appearing seeds and reduced seeds—the reduced seed being similar to those that generated the initial hybrids. It is likely that some of the normal (‘good’) seeds gave rise to the self-hybrids observed in the second cycle of hybridization (Table 4), although definitive conclusions cannot be drawn from the available data. In these pollinations, the development of the interspecific hybrid seed may have benefitted from the sharing fruit with the non-hybrid seed in what may be essentially a mentor pollen effect [19,20,21]. In these second cycle crosses, ‘Johnblue’ appeared as the better female parent, and it was perhaps fortuitous that the initial iteration of this cross had been performed with ‘Johnblue’.

The evaluated F_1_, US 2535-A, expressed only limited fertility as either male or female. This is not unlike other observations in diploid intersectional hybrids [10,22,23]. The hybrid itself had no apparent self-fertility. The lack of self-fertility is almost certainly due to the combination of low female fertility, low male fertility, and low self-compatibility. The production of a few seeds when using the hybrid as female, however, gives optimism for its future use. As a female, US 2535-A had a success rate of 0.01 seed/pollination. Currently, we have two verified BC_1_ plants, one of parentage US 2535-A × *V. vitis-idaea* ‘Sanna’, the other of parentage US 2535-A × *V. darrowii* ‘Native Blue’. Although diminishingly few in number, the success of these two hybrid combinations almost certainly guarantees that more such plants can be generated. We believe that the 75:25 genetic compositions of these backcross seedlings will likely improve both the male and female fertility in this BC_1_ generation of plants. The *V. vitis-idaea* backcross offspring are likely to allow the recovery of enhanced lingonberry types, with significantly improved heat adaptation and improved root-rot resistance. The backcrosses to *V. darrowii* may prove useful in bridging this material to 4*x* cultivated *V. corymbosum*.

## 4. Materials and Methods

### 4.1. Plant Materials

The genotypes used and their origins are listed in Table 6. All plants used were clones, and three to four plants of each genotype were used. Pollinations were performed on plants approximately five years old growing in 3 L pots in a 50:50 peat:sand mixture. All pollinations were performed in an insect-free greenhouse.

### 4.2. Crossing

Initially, *V. darrowii* ‘Johnblue’ × *V. vitis-idaea* ‘Red Sunset’ crosses were conducted, and based on the success of this cross, other cultivars from each species were selected, and additional crosses were attempted to corroborate the cross-compatibilities (Table 6). For all materials, pollen was extracted from open flowers by manual manipulation and collected on glassine weighing paper. Pollen was stored for up to a month under refrigerated and desiccated conditions until used for pollination.

Because of the small and somewhat delicate nature of the flowers on many of the parents, no emasculation was used. To perform pollinations, a graphite pencil tip was dipped into the collected pollen and applied to the stigmas of unemasculated flowers in an insect-free greenhouse. Pollinations were made on mature open flowers, and it was presumed that hybrids would be morphologically recognizable.

### 4.3. Stylar Examinations

To determine the viability of crosses, whole flowers with attached styles from experimental crosses were removed two days after pollination, using pollen as previously described, and fixed in a 3:1 solution of ethanol:acetic acid. After one week of fixation, the whole flowers were transferred to a solution of 90% ethanol until the date of examination. Styles were carefully detached from the remaining flower and stained using a 0.01% decolorized solution of aniline blue in 0.01 M potassium phosphate buffer. Upon preparation, the aniline blue solution was incubated at room temperature for 24 h to complete decolorization. To aid pollen tube visualization and facilitate aniline blue penetration of the inner stylar tissue, a tiny square 50% of the way down the style was delicately dissected on the stalk surface and meticulously removed so as not to disturb the inner contents of the style. An additional shallow and angled cut was made at the base of the style to detect pollen tubes that traveled the entire stylar length towards the floral ovary. After styles were whole-mounted in aniline blue solution and incubated for at least 2 h, slides were examined under ultraviolet light (excitation = 330–385 nm; emission = 420 nm), and tissue was imaged using an Olympus BX60 epifluorescence microscope (Olympus, Shinjuku, Tokyo, Japan) under its 10× objective, with Olympus cellSens Standard software (v1.11, core v3.10, Build 12201) (Olympus, Shinjuku, Tokyo, Japan). The examination of at least 3 styles per cross was determined to be sufficient for the clear assessment of cross success or failure, given that very little variation was observed within stylar samples.

### 4.4. Ploidy Determination

For flow cytometry, sampled young leaf material (1 cm^2^/20 to 50 mg) together with leaf material of an internal standard with known DNA content (*Zea mays* L.) were chopped with a sharp razor blade in 0.5 mL of extraction buffer (CyStain PI absolute P buffer, catalog number 05-5502; Partec, Münster, Germany) containing RNAse, 0.1% dithiothreitol (DTT), and 1% polyvinylpyrrolidone (ice cold) in a plastic petri dish. After 30 to 60 s of incubation, 2.0 mL staining buffer (CyStain PI absolute P buffer) containing propidium iodide (PI) as a fluorescent dye, RNAse, 0.1% DTT, and 1% polyvinylpyrrolidone was added. The sample, containing cell constituents and large tissue remnants of the chopped leaves, was then filtered through a 50 mm mesh nylon filter. After an incubation of at least 30 min at room temperature, the filtered solution with stained nuclei was measured with the flow cytometer (CyFlow ML (Partec) with a green diode laser 50 mW 532 nm (for use with PI); software: Flomax Version 2.4 d (Partec)). The DNA amount of the unknown samples was calculated by multiplying the DNA amount of the internal standard with the DNA ratio of the relative DNA amount of the unknown sample and the internal standard. DNA amounts were measured and compared to a set of standards covering the diploid to hexaploid range (2*x V. darrowii* ‘Fla 4B’*,* 4*x V. corymbosum* cv. Duke, and 6*x V. virgatum* cv. Powderblue) to determine basic ploidy levels.

### 4.5. DNA Analysis

In order to substantiate the hybridity of US 2535-A and the remaining hybrids with additional molecular information, the progenitors and putative hybrid seedlings underwent a paternity analysis by amplification of polymorphic simple sequence repeats (SSRs). The progenitor plants and putative hybrid seedlings were grown in a tunnel greenhouse at the P.E. Marucci Center for Blueberry & Cranberry Research in Chatsworth, NJ, in 2022 and 2023. Live cuttings were sent in 2023 by overnight mail to the USDA Cranberry Genetics and Genomics Laboratory (CGGL) in Madison, WI, for genetic testing. At CGGL, plant leaf tissue was freeze-dried using a BenchTop lyophilizer (Virtis, Gardiner, NY, USA), and DNA was extracted from dried leaf material.

A total of 0.03–0.04 g of freeze-dried leaf tissue per plant sample was pulverized to facilitate DNA extraction via a modified CTAB method [28] with added beta-mercaptoethanol (2 µL in 750 µL CTAB) and incubation at 65 °C for 1 h. Solubilized DNA from each plant sample was retrieved from an aqueous layer after adding 500 µL of chloroform with isoamyl alcohol solution in a 24:1 ratio and centrifuging at 14,000 rpm for 6 min. DNA was precipitated by adding cold isopropanol to the aqueous layers and placing in a freezer overnight at −20 °C. The next day, the DNA solutions were centrifuged at 14,000 rpm for 22 min to form a DNA pellet. Each pellet was washed twice in cold 70% ethanol, then resuspended in 50 µL of 1× TE buffer (10 mM TrisHCl pH 8.0, 1 mM EDTA pH 8.0) plus 3 µL of RNase-A. The final DNA in 1× TE plus RNase-A was incubated for 3 h at 36 °C, then transferred to 4 °C until use.

For this study, a subset of SSR markers originally developed for *Vaccinium macrocarpon* [29] and shown to be cross-transferrable across *Vaccinium* [14] were systematically tested on the progenitor DNA until 6 sufficiently polymorphic SSR markers were found for determining the paternity of our putative hybrids. Polymerase chain reactions (PCRs) were assembled in duplicate for each plant sample, in a total reaction volume of 8 µL per sample. Each reaction was comprised of 5 µL 1× JumpStart REDTaq ReadyMix (Sigma, St. Louis, MO, USA), 1 µL of plant DNA in 1× TE buffer, 0.5 µL of Betaine PCR Reagent (5M, MilliporeSigma), 0.5 µL of 5 µM hexachlorofluorescein (HEX) M13 primer, 0.5 µL of 5 µM forward SSR primer appended with the M13 5′-CACGTTGTAAAACGAC-3′ sequence, and 0.5 µL of 50 µM reverse SSR primer appended with 5′-GTTTCTT-3′. The modifications to the SSR forward [30] and reverse [31] primers serve to facilitate fluorescent labeling of PCR fragments and promote non-templated adenylation, respectively.

PCR was completed on S1000 Thermal Cyclers (Bio-Rad, Hercules, CA, USA) on a program of a single melting step at 94 °C for 3 min, followed by 33 cycles of 94 °C, 55 °C, and 72 °C for 15 s, 90 s, and 2 min, respectively—and a final extension step at 72 °C for 30 min. In total, 1 µL of each PCR reaction was added to 10 µL of a pre-mixed solution of formamide and carboxy-X-rhodamine ladder (Custom MapMarker ROX 75-375bp, BioVentures, Murfreesboro, TN, USA) at a ratio of 1000 µL:25 µL. The PCR–formamide samples were sent to Functional Biosciences, Inc. in Madison, WI, USA, for fragment analysis. Samples were run on an Applied Biosystems 3730 fluorescent sequencer with a 50 cm capillary array. The raw results were sent back to the CGGL, and genotyping calls were determined using GeneMarker software version 1.91 (Soft-Genetics LLC, State College, PA, USA). 

DNA fragment sizes (in bps) were determined at each marker for every plant in the study, including parentals and putative offspring (Table 1 and Table 4). At most, two distinct fragments were observed for a single marker, in agreement with the expectation that all plants in this study are diploid. The 6 markers used to determine hybridity were the following: SCF275d, SCF804, SCF9815, SCF37628, SCF132922, and 172672K70—whose sequences were previously provided [14]. All 6 SSR markers amplify polymorphic loci on separate linkage groups, according to Schlautman et al. [29].

### 4.6. Female Fertility

Cross numbers varied depending on flower availability. Pollinations and fruit set were recorded. Fruits were collected when ripe and measured for fruit size (mm in diameter) at the time of seed extraction. Extraction was performed manually under a dissecting microscope, and the seeds were evaluated for number and quality. For our purposes, seeds were classified as good, good-fair (g-f), fair, fair-poor (f-p), poor, or aborted. ‘Good’ and ‘fair’ described seeds that subjectively ranged from those considered fully normal to those somewhat reduced in size and/or development, but nonetheless were judged likely to be capable of germination. ‘Poor’ described seeds that displayed reduced size and/or development, were often flattened or brown, and were judged less likely to be capable of germination. Intermediate ratings were used as needed. ‘Aborted’ seeds were those that were flat and brown and generally translucent. Subjective observations were made on the size and quality of aborted seeds.

All extracted seeds were germinated directly on a greenhouse mist bench in a soil mix composed of 50:50, peat:sand mixture. At approximately a three true-leaf stage, seedlings were transplanted to 36-cell flats. Subsequently, plants were transferred to larger pots as appropriate for their growth stage.

### 4.7. Male Fertility

Pollen samples were stained with acetocarmine jelly (75% acetic acid with iron acetate) prepared according to the recipe of Jensen [32]. Pollen samples were assayed for quantity, stainability, and general condition. A microspore was considered potentially viable if it hydrated to a plump configuration and stained. Pollen was evaluated to determine to what degree perfect tetrads or defective tetrads were formed.

## 5. Conclusions

Crosses of *V. darrowii* ‘Johnblue’ × *V. vitis-idaea* ‘Red Sunset’ produced numerous true hybrids confirmed by molecular markers. The most vigorous and advanced of these hybrids was floriferous and morphologically intermediate to the respective parents. Examination of pollen suggested low male fertility, and numerous crosses using the hybrid as a female reflected similar low fertility and potential stylar crossing barriers. Nonetheless, two confirmed hybrid offspring have been produced using the F_1_ hybrid as a female in crosses using *V. vitis-idaea* and *V. darrowii*, respectively. Even if these backcrosses of our hybrids to the species’ parents prove to be infertile, these hybrids suggest other avenues that may exist for lingonberry improvement. If hybrid diploid seed duplicating our initial cross can be consistently produced in sufficient quantity, colchicine treatment might be effectively used to produce amphidiploid/allotetraploid plants. Ehlenfeldt et al. [4] have recently shown the feasibility of breeding such lingonberry hybrids at the tetraploid level using natural 4x hybrids. Alternatively, tissue culture could be used to somatically double US 2535-A and other similar superior genotypes. In either case, we believe such hybrids will find use in improving *Vaccinium* germplasm. Hybrids such as the one presented offer the potential for generating de novo interspecific fruit types in blueberry and/or broadening the adaptation of lingonberry.

## Figures and Tables

**Table 1:** DNA flow cytometry values for F_1_ hybrids and parents.

Genotypes	DNA Content (pg)
Ploidy standards	
2*x V. darrowii* ‘Fla 4B’	1.15
4*x V. corymbosum* ‘Duke’	2.08
6*x V. virgatum* ‘Powderblue’	3.51
	
F_1_ hybrids and parents	
2*x V. darrowii* ‘Johnblue’	1.10
2*x V. vitis-idaea* ‘Red Sunset’	1.05
F_1_ ‘Johnblue’ × ‘Red Sunset’ hybrids	
mean (*n* = 31)	1.09
std. dev.	0.037
range	0.99–1.17

**Table 2:** Simple sequence repeat (SSR) marker panel and DNA fragment sizes (in bps) after separation of PCR products by capillary electrophoresis observed in *Vaccinium* hybrids of *V. darrowii* × *V. vitis-idaea* (F_1_) and for backcross hybrids (BC_1_) of US 2535-A × *V. darrowii* and US 2535-A × *V. vitis-idaea*.

Genotype	SCF275d	SCF804	SCF9815	SCF37628	SCF132922	172672K70
*V. darrowii* ‘Johnblue’ (JB)	137, 151	222, 226	179	255, 268	186, 188	335, 361
*V. vitis-idaea* ‘Red Sunset’ (RS)	151, 169	244	187, 189	253	169, 171	331, 333
*V. darrowii* ‘Native Blue’ (NB)	133, 151	224, 230	179	263	174, 186	335, 381
*V. vitis-idaea* ‘Sanna’ (SAN)	171, 173	223, 225	187, 195	253	176	331, 333
US 2535-A (JB × RS clone A)	137, 151	222, 244	179, 187	253, 268	169, 186	333, 335
Johnblue × Red Sunset (F_1_) hybrids						
US 2535-A	137, 151	222, 244	179, 187	253, 268	169, 186	333, 335
#1	137, 151	222, 244	179, 187	253, 268	169, 186	333, 335
#2	137, 151	226, 244	179, 189	253, 255	171, 186	333, 335
#3	151	226, 244	179, 189	253, 255	169, 186	333, 335
#4	137, 151	226, 244	179, 189	253, 268	169, 186	331, 335
#5	137, 151	226, 244	179, 189	253, 255	169, 188	333, 361
#6	151	226, 244	179, 187	253, 255	169, 188	333, 361
#7	151, 169	226, 244	179, 187	253, 268	171, 188	333, 361
#8	151	222, 244	179, 189	253, 255	171, 186	331, 335
#11	137, 151	222, 244	179, 187	253, 268	169, 186	333, 335
#12	151, 169	222, 244	179, 189	253, 268	169, 188	331, 361
#13	137, 151	226, 244	179, 187	253, 268	171, 186	331, 361
#14	137, 151	226, 244	179, 189	253, 268	169 *	331, 335
#15	137, 169	222, 244	179, 187	253, 255	171, 188	331, 361
#17	137, 151	226, 244	179, 187	253, 255	171 *	333, 335
#19	151	226, 244	179, 189	253, 268	171, 186	331, 335
#21	151	222, 244	179, 189	253, 255	169, 188	331, 335
#22	151	226, 244	179, 189	253, 268	171, 186	331, 361
#24	151, 169	226, 244	179, 189	253, 268	169, 188	331, 335
#25	151	226, 244	179, 189	253, 268	169, 188	331, 335
#26	137, 151	226, 244	179, 189	253, 255	169 *	331, 361
#27	137, 151	222, 244	179, 187	253, 268	169, 188	331, 335
#28	151, 169	226, 244	179, 187	253, 268	169, 186	331, 335
#29	151, 169	226, 244	179, 187	253, 255	171, 188	331, 361
#30	137, 151	226, 244	179, 187	253, 268	171, 188	331, 335
#31	137, 169	226, 244	179, 187	253, 255	171, 188	333, 361
#33	151	222, 244	179, 187	253, 255	169 *	F

Freeze-dried leaf material (Genotype) was tested against six cross-transferable SSR primers (SCF275d, SCF804, SCF9815, SCF37628, SCF132922, and 172672_K70). Primer sequences are described in Rodriguez-Bonilla et al. 2019. The values denote PCR fragment weights (in bps). The parental DNA fragment values in the first two rows are necessary to determine parentage in the progeny. ‘F’ denotes reaction failure. * The fragment value does not match expectation; may be interpreted as a dropped allele when markers elsewhere show hybridity.

**Table 3:** Crosses of US 2535-A as a female.

							Fruit						
							Diameter	Seed Quality					
Pedigree					Pollinations.	# Fruit	(mm)	G	G-F	F	F-P	P	Seed/Pollination ^z^
US 2535-A	×	US 2535-A			97	5	7–8	0	0	0	0	0	0.0
											
US 2535-A	×	2*x*	*V. darrowii*	‘Johnblue’	24	5	6–6.5	0	0	0	0	1	0.0
”	×	”	”	‘Native Blue’	24	3	7.5–8.5	0	1	0	2	0	0.04
													
”	×	2*x*	*V. vitis-idaea*	‘Red Sunset’	53	12	5–8.5	1	0	0	0	0	0.02
”	×	”	”	‘Erntedank’	51	5	7–8	0	0	0	0	1	0.0
”	×	”	”	‘Sanna’	12	1	8.5	0	0	1	0	0	0.08
		**Total**			**261**	**31**		**1**	**1**	**1**	**2**	**2**	**Avg. = 0.01**

^Z^ Only seeds that were rated as ‘good’, ‘good-fair’, or ‘fair’ were counted as viable seeds.

**Table 4:** Crosses of *V. darrowii* × *V. vitis-idaea* genotypes.

									Fruit								
									Diameter		Seed Quality				Seed/	F_1_	F_1_/
Pedigree							Pollinations	Fruit	(mm)	G	G-F	F	F-P	P	Pollination ^z^	Hybrids	Seed
2*x*	*V. darrowii*	‘Fla 4B’	×	2*x*	*V. vitis-idaea*	‘Red Sunset’	25	13	5–8.5	0	5	12	0	0	0.7	1	0.06
																	
2*x*	*V. darrowii*	‘Johnblue’	×	2*x*	*V. vitis-idaea*	‘Red Sunset’	21	13	6–8	1	48	4	0	0	2.5	3	
”	”	”	×	”	”	‘Magenta’	13	13	5–8	5	39	0	2	0	3.4	7	0.07
”	”	”	×	”	”	‘Sanna’	11	11	5–8	6	32	1	0	0	3.5	0	
																	
2*x*	*V. darrowii*	‘Native Blue’	×	2*x*	*V. vitis-idaea*	‘Red Sunset’	11	10	6–11.5	16	0	2	0	0	1.6	3	
”	”	”	×	”	”	‘Magenta’	13	13	6–8	2	1	0	0	1	0.2	2	0.32
”	”	”	×	”	”	‘Sanna’	12	11	6–8.5	16	3	0	0	0	1.6	8	
				**Total**			**106**	**84**		**46**	**128**	**19**	**2**	**1**	**Avg. = 1.8**		

^Z^ Only seeds that were rated as ‘good’, ‘good-fair’, or ‘fair’ were counted as viable seeds.

**Table 5:** Simple sequence repeat (SSR) marker panel and DNA fragment sizes (in bps) after separation of PCR products by capillary electrophoresis observed in hybrids of *Vaccinium darrowii* × *V. vitis-idaea*, reciprocal crosses, and recapitulated F_1_ hybrids using alternate varieties of each species.

Genotype	SCF275d	SCF804	SCF9815	SCF37628	SCF132922	172672K70
*V. darrowii* ‘Fla 4B’	141, 151	222, 224	179	255, 263	174, 186	361, 381
*V. darrowii* ‘Johnblue’	137, 151	222, 226	179	255, 268	186, 188	335, 361
*V. darrowii* ‘Native Blue’	133, 151	224, 230	179	263	174, 186	335, 381
*V. vitis-idaea* ‘Red Sunset’	151, 169	244	187, 189	253	169, 171	331, 333
*V. vitis-idaea* ‘Magenta’	171, 173	223, 225	187, 195	253	176	331, 333
*V. vitis-idaea* ‘Sanna’	171, 173	223, 225	187, 195	253	176	331, 333
US 2535-A × Native Blue (BC_1_)	151	222, 230	179	263, 268	174, 186	333, 335
US 2535-A × Sanna (BC_1_)	137, 171	222, 225	179, 195	253, 268	186 *	333, 335
Fla 4B × Red Sunset (F_1_)						
US 2649	151, 169	224, 244	179, 189	253, 263	171, 186	333, 361
Johnblue × Red Sunset (F_1_)						
US 2650-1	151, 169	226, 244	179, 187	253, 268	169, 188	331, 361
(self) **	137, 151	222	179	255	186, 188	F
US 2650-2	137, 169	226, 244	179, 187	253, 255	186 *	333, 335
Johnblue × Magenta (F_1_)						
US 2651-1	137, 173	223, 226	179, 195	253, 268	188 *	331, 335
(self) **	137	222, 226	179	255, 268	186	335
US 2651-2	151, 173	226 *	179, 195	253, 268	176, 188	331, 361
US 2651-3	137, 171	222 *	179, 195	F	F	F
US 2651-4	137, 171	222, 223	179, 187	253, 268	176, 188	333, 335
US 2651-5	151, 171	223, 226	179, 187	253, 255	176, 188	331, 361
(self) **	137, 151	222, 226	179	255	186, 188	361
US 2651-6	137, 171	226 *	179, 187	253, 268	188 *	331, 361
US 2651-7	151, 171	222, 225	179, 187	253, 268	176, 186	333, 361
(self) **	F	226	179	F	F	F
(self) **	137	226	179	255, 268	186, 188	335
Johnblue × Sanna (F_1_)						
(self) **	151	222	179	255, 268	188	335
(self) **	137, 151	226	179	255, 268	186	335
Native Blue × Red Sunset (F_1_)						
US 2652-1	133, 151	224, 244	179, 189	253, 263	171, 174	331, 335
US 2652-2	133, 151	230, 244	179, 189	253, 263	171, 174	333, 381
US 2652-3	133, 169	224, 244	179, 189	253, 263	169, 186	331, 381
Native Blue × Magenta (F_1_)						
US 2653-1	133, 173	223, 230	179, 195	253, 263	176, 186	331, 335
US 2653-2	133, 171	225, 230	179, 187	253, 263	174, 176	331, 335
Native Blue × Sanna (F_1_)						
US 2654-1	133, 173	224 *	179, 195	F	186 *	333, 335
US 2654-2	151, 171	224 *	179, 195	253, 263	174 *	333, 381
US 2654-3	133, 171	223, 230	179, 195	253, 263	174 *	331, 335
US 2654-4	133, 171	225, 230	179, 195	253, 263	186 *	331, 381
US 2654-5	151, 173	225, 230	179, 195	253, 263	174 *	331, 381
US 2654-6	151, 173	223, 230	179, 187	253, 263	186 *	331, 381
US 2654-7	133, 173	223, 230	179, 195	253, 263	186 *	333, 381
US 2654-8	151, 173	224 *	179, 195	253, 263	174 *	331, 381

Freeze-dried leaf material (Genotype) was tested against six cross-transferable SSR primers (SCF275d, SCF804, SCF9815, SCF37628, SCF132922, and 172672_K70). Primer sequences are described in Rodriguez-Bonilla et al. [14]. The values denote PCR fragment weights (in bps). The parental DNA fragment values in the first four rows are necessary to determine parentage in the progeny. ‘F’ denotes reaction failure. * The fragment value does not match expectation; may be interpreted as a dropped allele when markers elsewhere show hybridity. ** Self of mother plant.

**Table 6:** Plant genotypes utilized in hybridization experiments.

Species and Genotype	Source [Reference]
*V. darrowii* (2*n* = 2*x* = 24)	
‘Johnblue‘	NC ARS and USDA [24]
‘Everblue’	NC-ARS and USDA [24]
‘Native Blue’	USDA [25]
Fla 4B (Florida 4B)	Draper and Hancock [2]
*V. vitis-idaea* (2*n* = 2*x* = 24)	
‘Red Sunset’	Hartmann’s Plant Co., Lakota, MI selection
’Erntedank’	Zillmer [26]
‘Sanna’	Sweden (GRIN Global) [27]
‘Magenta’	Swedish University of Agricultural Sciences

## Data Availability

The original contributions presented in the study are included in the article material, further inquiries can be directed to the corresponding author.
